# Reverse augmentation technique in hip revision arthroplasty: a new strategy for the management of acetabular reconstruction

**DOI:** 10.1186/s13018-020-01870-9

**Published:** 2020-09-10

**Authors:** Christian Götze, Christian-Dominik Peterlein

**Affiliations:** Clinic for Orthopedics, Auguste-Viktoria Clinic, Ruhr University Bochum, Am Kokturkanal 2, 32545 Bad Oeynhausen, Germany

**Keywords:** Acetabular defect, Augmentation technique, Restoration of the center of rotation

## Abstract

**Background:**

The principle of acetabular total hip revision (THR) is based on acetabular reconstruction and restoration of the center of rotation. The use of augmentation in high cranial acetabular defects combined with a cementless revision shell was studied sufficiently. This study aimed to report a case with the use of an augment inside a cementless revision shell as a reverse augmentation technique.

**Methods:**

We describe the case of an 86-year-old female patient with a massive acetabular defect during second revision for total hip arthroplasty (THA). Two problems occurred: (1) a fixed cemented stem with a nonmodular head size of 33 mm and (2) a high acetabular defect with an elevated rotation center.

**Results:**

With the distraction technique, allograft filling was used to reconstruct the acetabular defect. A cementless revision shell (REDAPT, Smith and Nephew) with a size of 78 mm was used to stabilize the defect. Locking screws placed cranially and distally were used to stabilize the cup for secondary osseointegration. An augment was placed inside the cup to reconstruct the rotation center. A customized polyethylene liner (outer diameter, 54 mm/inner diameter, 33 mm) was positioned below the augment in the revision cup to reconstruct the center of rotation. An 18-month postoperative X-ray analysis showed a stable construct with full secondary osseointegration.

**Conclusion:**

This is the first report of an augment used for a reverse technique inside a cementless shell to restore the center of rotation with the use of a customized polyethylene liner. This might be a reliable option for reconstruction of the center of rotation in large cementless revision cups in acetabular Paprosky type III defects. This technical note shows the possibility of using an augment as a reverse technique in a cementless revision cup.

## Background

Acetabular revision arthroplasty is based on three main principles. The first is the primary stable fixation of the revision cup in the acetabular defect after second principle, which is bony reconstruction. The third principle is the restoration of the center of rotation to avoid instability and to maintain long-term stability. Different implant designs and sizes are available for THA acetabular revisions [1]. In the last decade, a trend towards cementless revision systems has occurred [2]. With regard to the extent of acetabular defects, bony allograft reconstruction or metal augmentation seems to be the choice for addressing major bone loss [3]. Different studies present sufficient results of acetabular metal augmentation [4–7]. In principle, the augment is used to reconstruct the cranial defect while positioning the augment above the cementless cup. We describe the case of a patient with an augment placed inside a cementless shell to reconstruct the center of rotation. To the best of our knowledge, no study has reported this technique to date.

## Case

This case report aimed to reconstruct a highly acetabular defect in an 86-year-old female patient. The first cemented THA was performed in the patient in 1973 at the age of 41 years due to secondary osteoarthritis in regard to hip dysplasia. A nonmodular stem combined with a fixed 33-mm head (Fa. Link) was used as the primary femoral component.

In 1998, more than 20 years after the primary arthroplasty, loosening of the cup was detected. A hip revision arthroplasty was performed with exchange of the acetabular component. An oblong revision cup was implanted to stabilize the defect. Inside the cup, a custom-made implant with an inner diameter of 33 mm was cemented into the acetabular cementless revision component.

**Fig. 1:**
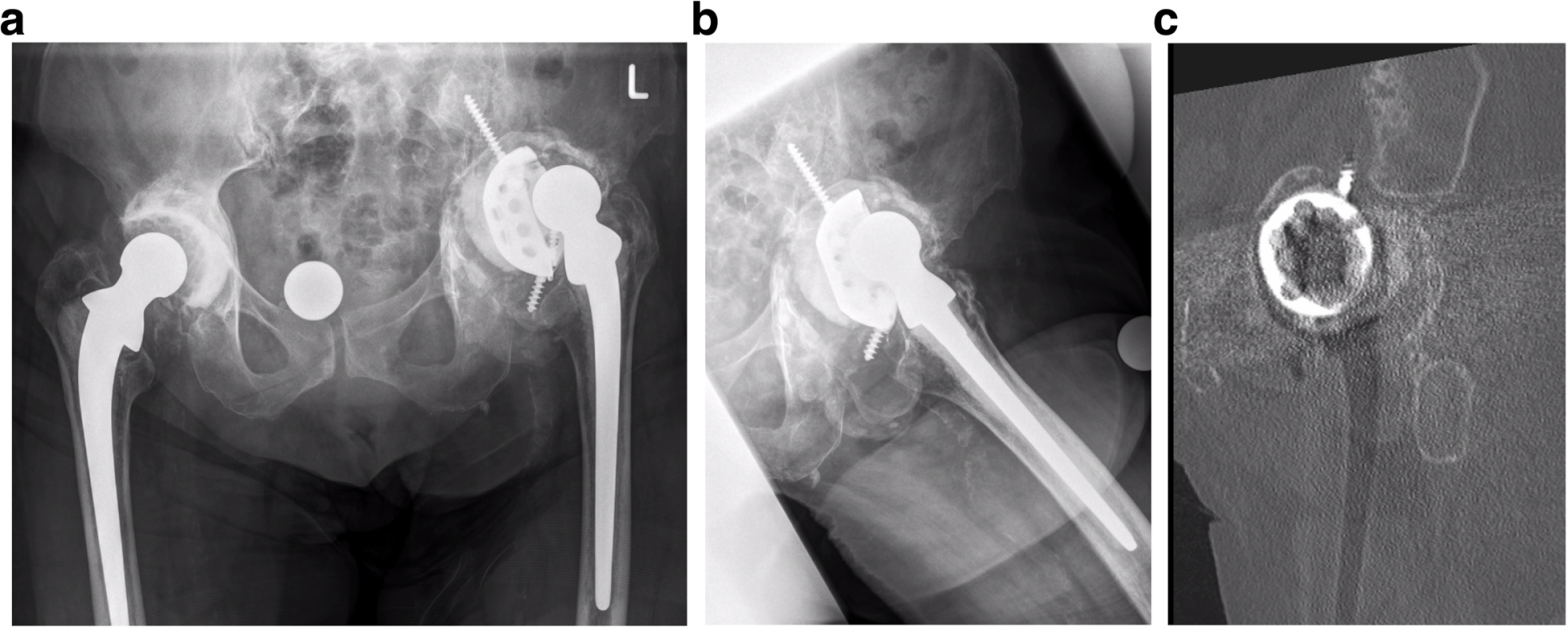
**a** X-ray of an 86-year-old woman who underwent first THA with a monobloc stem and a 33-mm head (Fa. Link) in 1973. In 1998, revision arthroplasty was performed with an oblong revision cup (LOR) for a high defect on the acetabular side with a well-fixed cemented stem. **b** Acetabular defect with a high rotation center. **c** CT data of the acetabular defect correlated with pelvic discontinuity

Twenty years after the first revision arthroplasty, the patient presented with loosening of the acetabular component. A high acetabular defect classified as a type IIIb defect based on the Paprosky classification system was defined as an “up and in” defect with superior and medial migration of the femoral head > 3 cm (Fig. 1a). A lateral view radiograph (Fig. 1b) and computed tomography scan (Fig. 1c) demonstrated a high acetabular defect near an area of pelvic discontinuity. The cemented stem was still in place and did not demonstrate any signs of loosening 45 years after primary implantation.

Two main problems had to be considered in this revision procedure: first, the high acetabular defect with major bone loss, and second, the reconstruction of the center of rotation in this distinct case.

Revising the fixed stem in this elderly patient with a nonmodular stem would create a major femoral defect in this weak bone. With regard to this problem, a decision was made to create a custom-made polyethylene liner with an outer diameter of 54 mm and an inner diameter of 33 mm for the fixed head of the monobloc stem (Fa. Link). With this technique, revision of the fixed stem could be avoided.

**Fig. 2:**
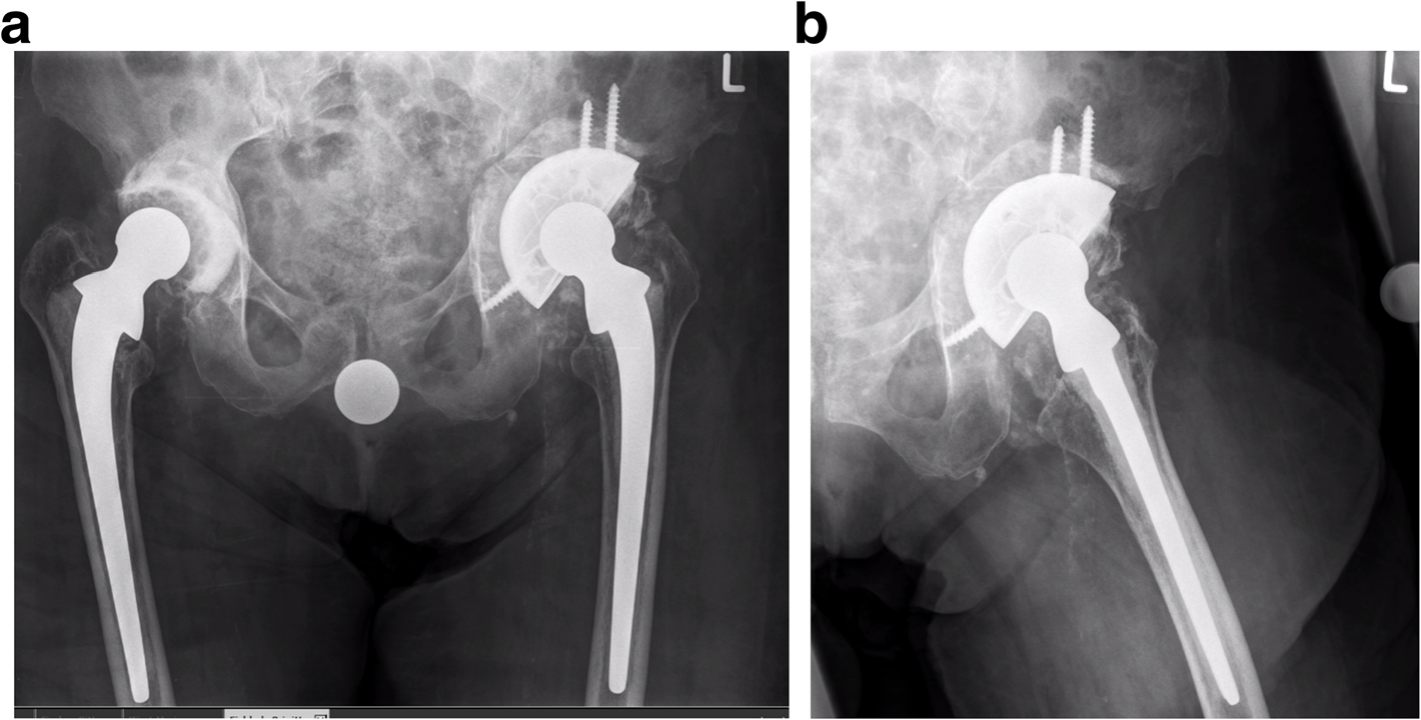
**a** Allograft filling with the distraction technique. The defect was stabilized with a cementless revision shell (REDAPT size, 78 mm, locking screws cranial and distal). Reconstruction of the center of rotation with the reverse augmentation technique. **b** Lateral view of the postoperative X-ray

The operation was performed with the supine technique and a lateral approach. After removal of the loosed acetabular cup, based on the distraction technique of Sporer [8], we created a bony allograft construct to fill the defect. A cementless revision component REDAPT (78 mm) (Smith and Nephew Orthopedics AG, Rotkreuz, Switzerland) was placed into the acetabular defect as a primary stable construct. Two locking screws (size, 30/35 mm) were placed cranially in the bony structure, and two locking screws (25/30 mm) were placed distally into the os ischii to bridge the defect (Fig. 2a, b).

**Fig. 3:**
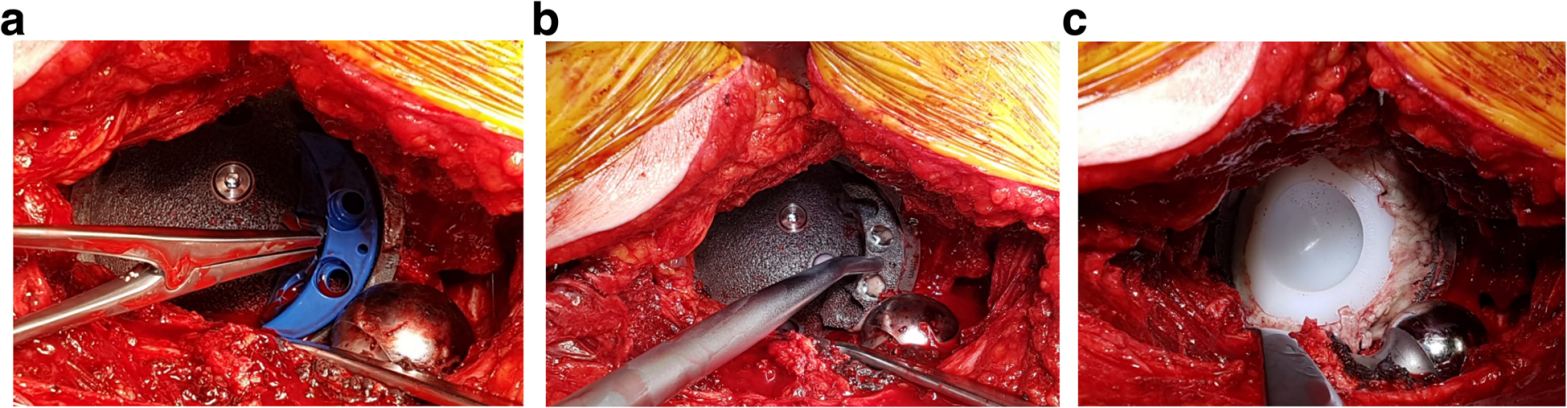
**a** Trial augment staple with a size of 62/12 mm in a cementless REDAPT cup with a size of 78 mm. **b** Augment staple with a size of 62/12 mm cemented in a REDAPT cup with a size of 78 mm as a reverse augmentation technique. **c** Customized polyethylene cup (54 mm, Fa. Link) for the nonmodular 33-mm head

An augment with a size of 62/12 mm was cemented cranially into the titanium shell to diminish the defect in the cranial–caudal dimension (Fig. 3b). The augment size was determined by the inner side of the revision cup. Underneath the augment, a custom-made polyethylene liner was cemented with a thin cement liner in the center of rotation (Fig. 3c). With this technique, tension of the THA was appropriate to avoid any dislocation. The duration of the operation was 120 min. The total blood loss was 500 ml, and the amount of blood transfusion was 2 units intraoperatively. The patient was mobilized with limited weight bearing for 6 weeks. There were no further postoperative complications.

**Fig. 4:**
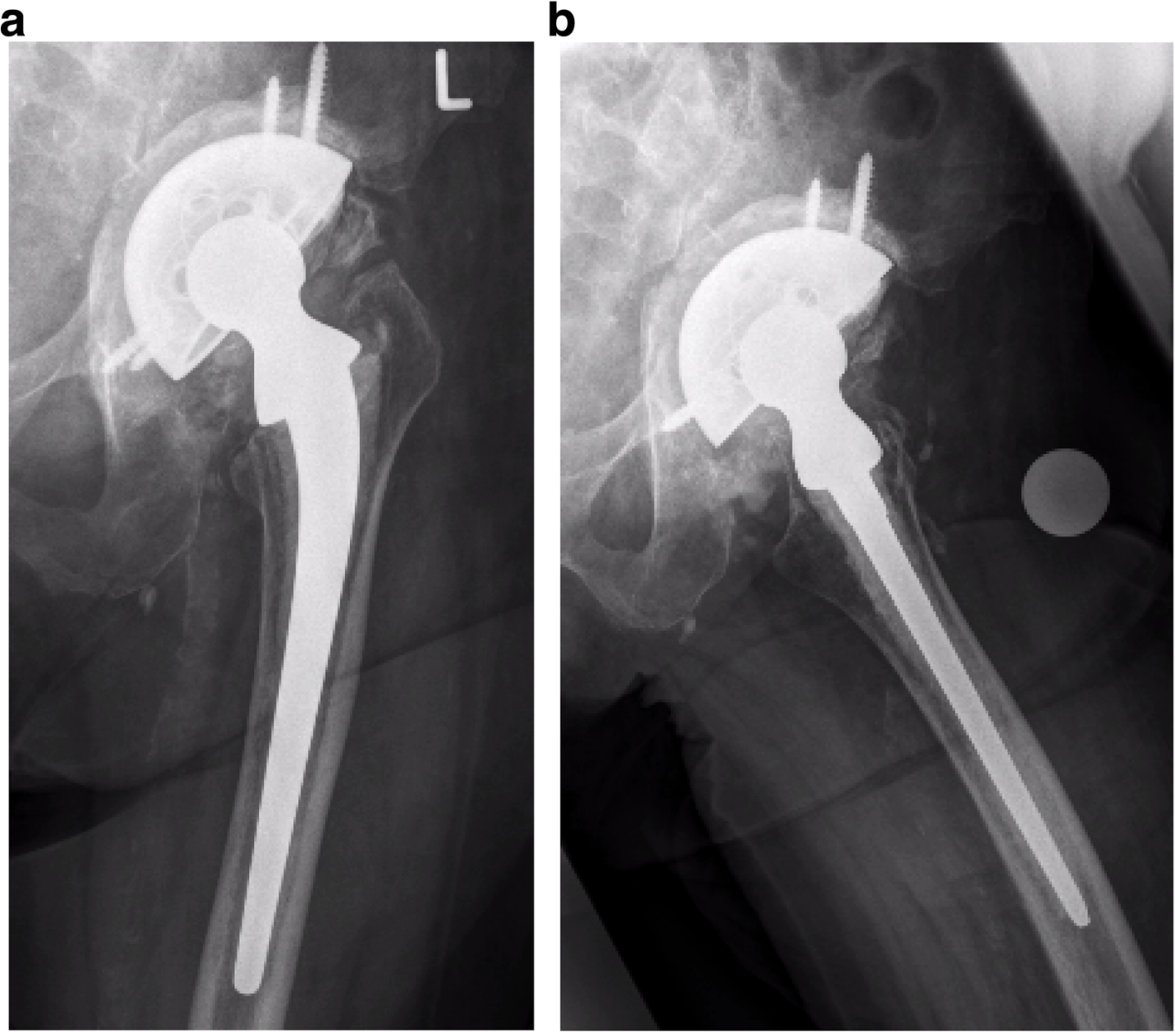
**a** The 18-month postoperative revision arthroplasty, with no signs of loosening of the acetabular component with secondary osseointegration. **b** Lateral view 18 months postoperatively

A total of 18 months after the revision arthroplasty, the patient was observed to have no signs of loosening of the THR with secondary osseointegration (Fig. 4a, b). The mega revision cup was still in place. The patient was quite well without any signs of pain in her affected left hip joint. Ambulation was sufficient with the help of one crutch on the contralateral side. The patient reported good quality of life and pain reduction with an increased walking distance. The VAS pain score decreased from 8 points to 2 points at the last follow-up. The HHS improved from 43 points preoperatively to 72 points 18 months postoperatively.

## Discussion

Based on the literature, this is the first case of an acetabular augment placed inside the cementless revision cup to adjust the center of rotation in combination with a custom-made polyethylene implant. To date, this is an off-label procedure to reconstruct this situation with a fixed monobloc stem and a 33-mm head in an 86-year-old female patient. At 18 months postoperatively, this combination was radiologically stable with sufficient clinical and functional results.

The use of modular acetabular revision systems with high porosity for complex defects has achieved increased attention in the last decade. Different studies have presented excellent short- to mid-term results in the current literature [4–7]. The advantage of most modular systems is based on the possibility of acting variably in different situations to restore acetabular defects. Augments of different sizes and heights can be placed cranially to restore acetabular defects. By using this technique, the defect will transform from oval to spherical in shape to reconstruct the center of rotation. With regard to the high porosity of the acetabular cup structure, primary stability will be enhanced [9]. Micromotion can be reduced, and osseointegration of the metal cup will be optimized. To date, even severe defects can be sufficiently solved [10]. Additional screws are used to stabilize the modular construct in the defect zone. Although the use of trabecular metal is more well known, fully porous titanium implants have gained increased attention. In this case, a titanium shell with a porosity of > 80% [11] was used to maintain stability. With the use of locking screws, the stability of the construct was enhanced even in severe conditions.

The REDAPT revision cup is based on the concept of additional locking screws for the cup and augment. With locking screws, in vitro analysis demonstrates higher stability. By placing the screws cranially and caudally, the revision shell can be placed securely even in higher acetabular defects or in cases of pelvic discontinuity after allograft augmentation. By placing the augment inside the cup, the rotation center of the fixed femoral head can be effectively repositioned. Reconstruction of the center of rotation might be the most important factor for long-term stability [12]. Similar to the problem in Jumbo cementless revision cups, the center of rotation is elevated with regard to the wide diameter of the shell. Modified hemispherical implant geometry can reduce head center elevation with favorable biomechanical conditions, as demonstrated by an in vitro analysis [13]. The only potential problem might be in the combination of the convex side of the augment with the concave side of the acetabular shell by a cement layer. The potential risk of failure in this modular acetabular reconstruction has not been described thus far in the literature.

## Conclusion

This is a technical note in acetabular revision arthroplasty. A custom-made implant was produced to avoid revision on a fixed stem with an unusual shaft head diameter. In a cementless mega revision cup, by reverse augmentation, the cemented custom-made cup was placed in the center of rotation. The augment was fixed with a thin cement layer. By using this technique, femoral head dislocation can be prevented, and long-term stability of the construct is secured. To date, this is an off-label reconstruction. At least mid-term results are necessary to confirm that this procedure is effective. We will continue to follow this case carefully.
